# Co-designing an exercise maintenance intervention for older adults exiting falls prevention programmes: a multi-stage stakeholder consultation approach

**DOI:** 10.1080/21642850.2026.2662110

**Published:** 2026-04-22

**Authors:** Sarah Audsley, Nicola Adams, Gill Barry, Paul Court, Seema Haridas, Rory Harrison, Amy Hemmings, Bob Laventure, Victoria Mercer, Sarah A. Moore, Alasdair F. O’Doherty, Emma Stanmore, Dawn A. Skelton

**Affiliations:** a School of Sport, Exercise and Rehabilitation, Health and Life Sciences, Northumbria University, Newcastle Upon Tyne, UK; b School of Health and Care Sciences, University of Lincoln, Lincoln, UK; c HealthWorks Community Health Charity, Newcastle Upon Tyne, UK; d The Newcastle Upon Tyne Hospitals NHS Foundation Trust, UK; e Later Life Training Ltd., Newcastle Upon Tyne, UK; f Stroke Research Group, Faculty of Medical Science, Newcastle University, Newcastle Upon Tyne, UK; g Research and Development, Northumbria Healthcare NHS Foundation Trust, North Shields, UK; h Department Sport & Exercise Science, Durham University, Durham, UK; i Division of Nursing, Midwifery & Social Work, The University of Manchester, Manchester, UK; j Research Centre for Health (ReaCH), Glasgow Caledonian University, Glasgow, UK

**Keywords:** Falls prevention, exercise maintenance, co-design

## Abstract

**Background:**

Falls Management Exercise (FaME) programmes improve physical function and reduce falls rate and risk in older adults, but after completion exercise engagement declines, leading to loss of the health benefits gained. This study aimed to co-design an exercise maintenance intervention for older adults exiting FaME programmes, addressing the critical need to sustain physical activity benefits after programme completion.

**Methods:**

A three-stage co-design process: Stage 1 involved community-based stakeholder consultations with older adults completing FaME and rehabilitation professionals (*n* = 16) to identify acceptable intervention components. Stage 2 consisted of professional stakeholder consultations (*n* = 11) to assess feasibility. In Stage 3, the Normalisation Process Theory guided development of implementation procedures with service-providers (*n* = 2). Data were analysed using descriptive statistics and thematic analysis.

**Results:**

Community-based stakeholders identified core components: information on local physical activity opportunities (88%), motivational strategies (81%), follow-up group meetings (69%), and health education (63%). Professional stakeholders confirmed unanimous agreement for motivational strategies and health education. Concerns emerged regarding affordability of follow-up meetings for service-providers and equity of digital interventions. Follow-up phone calls, text message reminders, self-completed functional fitness assessments and exercise snacks in face-to-face sessions emerged as additional components.

**Conclusion:**

KESS is a rigorous, co-designed, stakeholder informed intervention targeting exercise maintenance in older adults after FaME ends. KESS aims to prevent a decline in exercise adherence and consequent increasing risk of falling, observed in FaME programme attendees >6 months after programmes end if appropriate activity is not maintained. KESS is ready for feasibility and acceptability testing.

## Introduction

Falls in older adults represent a major public health challenge worldwide (Montero-Odasso et al., 2022). Globally, one in three adults aged over 65 years, and half of adults aged over 80 years, fall each year with one in five being classed as injurious falls (Ambrose et al., 2013; Tinetti et al., 1988). Falls impair quality of life (Montero-Odasso et al., 2022), yet meta-analyses show 24–42% of falls are preventable through strength and balance exercise interventions (Sherrington et al., 2020). Understanding the multifaceted nature of falls risk is essential for developing effective, sustainable prevention strategies.

Multiple factors increase falls risk, including social isolation, environmental hazards, medication effects, cognitive impairments, and poor muscle strength and balance (Petersen et al., 2020; Rubenstein, 2006). Physical inactivity, prevalent in older adult populations (Harvey et al., 2015), contributes to muscle weakness, poor balance, and recurrent falls (Cunningham et al., 2020). Strength and balance exercise performed for at least 2 hours per week over 6 months improves function and prevents falls (Sherrington et al., 2019). The Falls Management Exercise (FaME) programme is an evidence-based 6-month, group-based exercise intervention proven to reduce falls (Iliffe et al., 2014; James et al., 2022; Orton et al., 2021; Skelton et al., 2005; Skelton et al., 2019). However, physical activity (PA) typically declines 6–24 months after FaME programmes end (Iliffe et al., 2014; Orton et al., 2021), increasing falls risk (Orton et al., 2021; Sherrington et al., 2019). This post-programme decline represents a critical adherence gap undermining long-term effectiveness of falls prevention interventions.

Maintaining falls prevention behaviours post-intervention is a well-documented challenge across healthcare settings globally. Community-based programmes face persistent barriers to sustaining exercise engagement once structured support ends (Maula et al., 2019; Ventre et al., 2025). Hospital-based studies report parallel maintenance challenges, with intervention gains diminishing after discharge (Heng et al., 2020; Hill et al., 2015; McLennan et al., 2024). Research consistently identifies common barriers across diverse settings, including funding constraints, instructor capacity limitations, time pressures, and declining participant motivation post-intervention (Heng et al., 2020; Maula et al., 2019; McLennan et al., 2024; Ventre et al., 2025). Falls prevention research suggests that behaviour change strategies, education, social support, digital technologies, and transition support can encourage continued exercise engagement (Audsley et al., 2020; Bunn et al., 2008; Choi et al., 2021; Finnegan et al., 2019; Hawley-Hague et al., 2020; Hobbs et al., 2013; Maula et al., 2019; Morris et al., 2022). Despite the emerging evidence on strategies that may support maintenance, developing interventions that can be implemented and sustained in real-world settings remains challenging.

Traditional research-led intervention development approaches often fail to capture the practical constraints, priorities, and contextual factors necessary for successful real-world implementation (Yardley et al., 2015). Co-design approaches, which embed service-users and professional stakeholders within the intervention development process (Leask et al., 2019; Moll et al., 2020) are considered essential in falls prevention research to help address real-world needs (Grindell et al., 2022; McKercher et al., 2025; Morris et al., 2025). This study aimed to co-design an exercise maintenance intervention acceptable for FaME service-users and feasible for service-providers within real-world settings.

## Methods

### Study design

**Figure 1.**
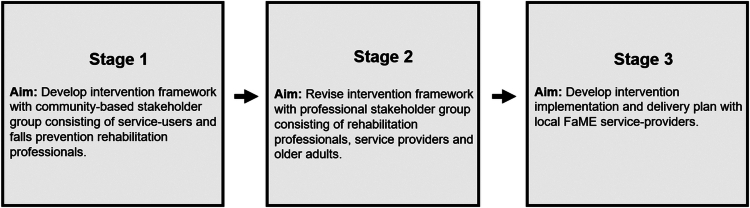
Overview of the intervention development stages.

A three-stage co-design process incorporated community-based and professional stakeholder group consultations, and implementation planning discussions with service-providers (Figure 1). The mixed-method approach integrated quantitative voting data with qualitative thematic analysis of round-table discussions to ensure authentic stakeholder involvement in developing a co-designed exercise maintenance intervention (Grindell et al., 2022).

### Theoretical frameworks

Self-efficacy, autonomy, and social support are key determinants of PA maintenance in older adults (Maula et al., 2019; McAuley et al., 2011). Therefore, the Social Cognitive Theory (SCT) and Self-Determination Theory (SDT) were selected to underpin the intervention development through their emphasis on the key determinants (Kwasnicka et al., 2016; Xu et al., 2025). To examine future implementation potential, the APEASE criteria (Acceptability, Practicability, Effectiveness, Affordability, Side-effects, Equality) guided intervention strategy evaluation in Stage 2 (Michie et al., 2014). NPT guided Stage 3 implementation planning at local service-provider level (May & Finch, 2009). Service-provider discussions focused on three key NPT domains: how the KESS intervention would fit within existing services (coherence), how staff could engage with the intervention (cognitive participation) and how the intervention could be delivered within available resources (collective action).

### Stage 1: community-based stakeholder group consultations

**Aim:** To identify acceptable intervention components for service-users to receive when FaME programmes end and develop an intervention framework.

**Stakeholders and recruitment:** Falls prevention exercise class attendees were recruited from commissioned community-based FaME programmes delivered by Healthworks, a community health charity within Newcastle-Upon-Tyne, UK. Eligible participants were those who had completed or would complete their FaME programme within one month of the planned workshop date and were able to provide informed consent. Healthcare professionals (HCPs) and postural stability instructors (PSIs, trained to deliver FaME) were identified through local falls prevention networks using snowball sampling and invited via email. Eligible professionals included those with experience delivering falls prevention services and able to give informed consent. All interested parties were provided with a participant information sheet and given the opportunity to ask questions prior to providing informed written consent.

**Procedures:** A two-hour face-to-face consultation was conducted at a Northeast leisure centre with service-users, HCPs and PSIs. Stakeholder characteristics (age, gender, ethnicity and technology use) were collected before consultations. The research team presented eight evidence-based intervention components derived from falls prevention (Audsley et al., 2020; Choi et al., 2021; Finnegan et al., 2019; Iliffe et al., 2014; Maula et al., 2019) and PA maintenance literature (Hobbs et al., 2013; McAuley et al., 2011) (Table 1). Each component was described verbally and, where applicable, demonstrated using example materials (e.g. KOKU app interface [https://kokuhealth.com/], printed booklet samples, fitness watch functionality). Audio-recorded round-table discussions explored the acceptability and effectiveness of each component. At the end of each discussion, stakeholders completed anonymous voting forms to rate components as definitely include, include as an option, or definitely exclude from the exercise maintenance intervention.

**Table 1.** Intervention components presented to community-based stakeholders.

Component	Description	Evidence source	Presentation method
Local PA information	Signposting to community exercise opportunities	Finnegan et al. (2019), Hobbs et al. (2013)	Verbal description with example leaflet
Motivational strategies	Goal setting, self-monitoring, reinforcement, relapse prevention	Hobbs et al. (2013), McAuley et al. (2011)	Verbal explanation with verbal and written examples
Follow-up group meetings	Post-programme meetups	Audsley et al. (2020), Maula et al. (2019)	Verbal description and written examples
Health education	Benefits of continued exercise and healthy eating	Finnegan et al. (2019)	Verbal presentation and written examples
Digital exercise support	KOKU digital intervention for guided home exercises	Choi et al. (2021)	Verbal description and live app demonstration and play
Printed exercise booklets	Illustrated home exercise guides	Audsley et al. (2020), Iliffe et al. (2014)	Verbal description and physical booklet samples
Fitness watches	Wearable activity monitors	Maula et al. (2019)	Verbal description and device demonstration
Peer connectivity	Strategies to maintain participant contact	Audsley et al. (2020), Maula et al. (2019), McAuley et al. (2011)	Verbal description with examples

PA, Physical Activity; KOKU app, Keep On Keep Up application.

**Data Analysis:** Voting data were analysed descriptively and organised hierarchically by ‘definitely include’ responses. A three-tiered traffic-light classification system was applied to visualise stakeholder consensus: high consensus (green) represented components with ≥60% of stakeholders selecting ‘definitely include’; moderate consensus (amber) indicated 25–59% agreement; and low consensus (red) reflected ≤24% agreement. Audio recordings were transcribed and analysed thematically using NVivo14 by two independent researchers. The purpose of the hierarchy was to quantify the level of agreement of each intervention strategy to create an intervention framework. Themes in the qualitative data were used to help understand nuances in the voting results and contextualise how each intervention strategy might be effectively implemented.

### Stage 2: professional stakeholder group consultations

**Aim:** To assess intervention feasibility using APEASE criteria and refine the framework for real-world delivery.

**Stakeholders and recruitment:** Professional stakeholders included HCPs, PSIs, service managers, a public health commissioner, a clinical academic, and older adult representatives all with experience of falls prevention. Stakeholders were identified by local service-providers through snowball sampling. Stakeholders were invited to contribute to co-design discussions via email. Interested parties were provided with a participant information sheet and given the opportunity to ask questions prior to giving informed written consent.

**Procedures:** Two-hour consultations were conducted online via Microsoft Teams. Researchers presented Stage 1 findings and the intervention framework. Stakeholders were asked to consider and evaluate each component specific aspects using the APEASE (Michie et al., 2014) criteria as follows: Acceptability (would service-providers and service-users find this appropriate?), Practicability (can it be delivered at scale?), Effectiveness (will it help older adults stay active?), Affordability (is it financially viable for service-providers and service-users?), Side-effects (any unintended consequences?), and Equality (would it increase or decrease inequalities?). Anonymous online voting forms assessing feasibility against all APEASE criteria were completed the day after consultations took place.

**Analysis:** Voting data were summarised descriptively according to APEASE criteria. For each intervention component, stakeholder agreement was assessed across all six APEASE criteria (Acceptability, Practicability, Effectiveness, Affordability, Side-effects, and Equality). A three-tiered traffic-light consensus classification was applied based on the total number of respondents (out of 10) who agreed across all APEASE criteria: high consensus (green) represented n ≥ 9–10 respondents agreeing across all six APEASE criteria; moderate consensus (amber) indicated *n* = 6–8 respondents agreeing across most criteria; and low consensus (red) reflected *n* ≤ 5 respondents agreeing across most criteria. Intervention components achieving ≥6/10 agreement on at least five APEASE domains were considered acceptable for inclusion.

Transcribed workshop discussions were analysed thematically (Braun & Clarke, 2006), with themes organised by intervention component. The qualitative analysis identified contextual factors influencing APEASE ratings and modifications that could improve acceptability.

### Stage 3: implementation planning

**Aim:** To refine the intervention into deliverable protocols with service-providers.

**Stakeholders:** Healthworks PSI and service manager.

**Procedures:** The intervention framework was organised into a draft schedule of activities (originally 6 sessions post-FaME), emailed to stakeholders and discussed in an online meeting. Discussions were guided by three Normalisation Process Theory domains (May & Finch, 2009). These explored: coherence (how KESS integrated with existing FaME delivery), cognitive participation (PSI willingness, training needs, and perceived programme value), and collective action (practical delivery considerations including time, resources, and workload). Fieldnotes documented schedule modifications, barriers and solutions.

**Analysis:** Fieldnotes were used to finalise the schedule of activities, develop the 2-day PSI KESS training programme, standard operating procedures and participant resources.

### Ethics statement

Ethical approval was obtained from Northumbria University’s Research Ethics Committee [30/01/2024, Reference: Audsley-2024-6365-5990] and study protocol registered on ClinicalTrials.gov [NCT06447948].

## Results

### Stage 1: community-based stakeholder group results

**Stakeholders:** In Stage 1, 18 individuals expressed interest (12 FaME class attendees, 6 professionals), with 16 attending the stage 1 consultation. Two individuals opted out due to scheduling conflicts and lack of interest. The 16 stakeholders who attended the Stage 1 consultation were: 10 FaME class attendees (median age 81 years, IQR 76-84 years, range 66-87 years; 9 female, 10 Caucasian) and 6 HCPs/PSIs (median age 40 years, IQR 33-47 years, range 23-47 years; 5 female, 6 Caucasian). Technology use was relatively high among the 10 FaME class attendees, with 9 owning smartphones, 6 owning tablets, and 7 using WhatsApp.

**Voting Results:** Voting responses revealed clear stakeholder preferences for intervention components. Results are presented in a three-tiered traffic-light hierarchical framework based on ‘definitely include’ responses (Table 2).

**Table 2.** Stage 1 KESS intervention framework based on community-based stakeholder results.

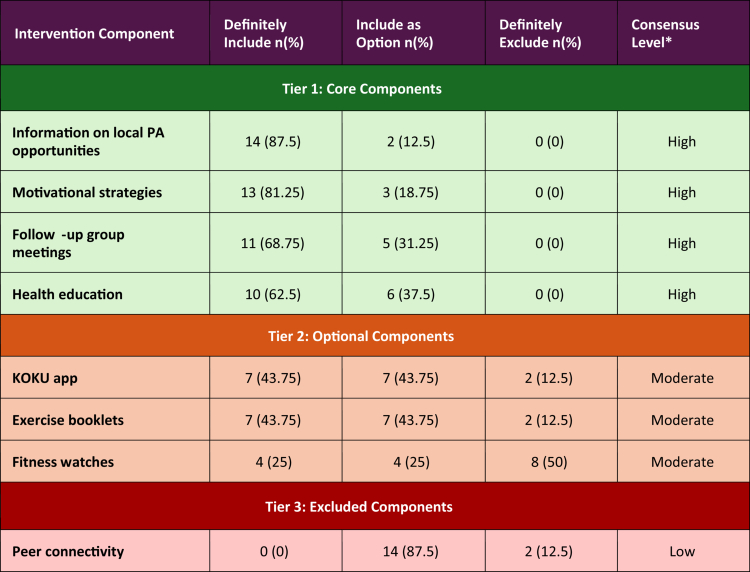

Three-Tiered Intervention Framework Model Based on Community Stakeholder Voting (*n* = 16). **Consensus classification**
High consensus: *n* = 10 to 16 of 16 stakeholders (≥60%). Moderate consensus: *n* = 4 to 9 of 16 stakeholders (25 to 56%). Low consensus: *n* = 0 to 3 of 16 stakeholders (≤24%).

### Thematic analysis

Stakeholder consultations revealed intervention-specific themes across 6 key areas, highlighting both barriers and facilitators for each component see Table 3 and supplementary material 1. The thematic analysis revealed cross-cutting themes of personalisation, accessibility, and the critical importance of promoting social connection within the intervention design.

**Table 3.** Thematic analysis and representative quotes outlining community-based stakeholder group discussions.

Themes	Quotes
**Information on local physical activity opportunities** received universal support, though stakeholders emphasised the need for current, accessible information including venue accessibility, cost, and age-appropriate options. Professionals noted significant resource requirements for maintaining up-to-date service directories.	*“If you haven't got all the information, you're not going to know where to go”* (R6_ Service-user) *“Keeping on top of that list would be a significant part of our job”* (R9_Professional)
**Motivational strategies** generated strong consensus for peer support, flexible goal setting, and routine development, with stakeholders emphasising the importance of contingency planning and avoiding rigid expectations that could lead to feelings of failure.	*“Not being too rigid in your goals... if you don't achieve it once, you're like, oh well, failed”* (R6_Service-user)
**Follow-up group meetings** received unanimous support from service-users primarily for social connection and loneliness reduction rather than exercise motivation, with emphasis on commitment and peer accountability.	*“I think it would be great, as you mentioned... Whether it would encourage me to exercise, I don’t know. But it would be nice to meet people.”* (R4_Service-user)
**Health education** was viewed positively but considered less effective than other components, with preferences for accessible delivery by PSIs rather than complex medical information.	*“My doctor tends to send me a ton of stuff that bamboozles me... I tend to ask my class instructor”* (R2_Service-user)
**Technology-based vs paper-based interventions for home exercise** (KOKU digital strength and balance exercise intervention (Choi et al., 2021), exercise booklets (LaterLife Training, 2024), fitness watches) revealed divided opinions largely dependent on digital literacy and experience, with consistent themes around the need for ongoing technical support and accessibility concerns.	*“If you had support … show you how to use it and if that (support) was ongoing”* (R5_Service-user) *“What's the kind of rules around sharing phone numbers... I can imagine that's a bit of a minefield”* (R9_Professional)

### Stage 2: professional stakeholder group results

**Stakeholders:** In Stage 2, 11 individuals were invited, and all (100%) attended a stage 2 consultation. The professional stakeholders who participated were falls prevention HCPs/PSIs (*n* = 7), clinical academics (*n* = 1), older adult representatives (*n* = 2), and public health commissioners (*n* = 1) (median age 46 years, IQR 44-55 years, range 27-76 years; 9 female, 11 Caucasian).

**APEASE Assessment Results:** 10 out of the 11 stakeholders completed the online voting forms; 1 stakeholder did not return their form for unknown reasons. Feasibility evaluation revealed distinct patterns of professional stakeholder agreement across intervention components (Supplementary material 2).

Eight intervention-specific themes emerged from stakeholder group discussions. The thematic analysis revealed how stakeholders reasoned through their APEASE evaluations. Cross-cutting themes included personalisation needs, resource sustainability concerns, and the importance of promoting individual choice within the intervention design, see supplementary material 3. Table 4 presents a three-tiered traffic-light consensus framework based on the level of agreement across all APEASE criteria and the key considerations arising from the thematic analysis that influenced ratings.

**Table 4.** Stage 2 KESS intervention framework, based on the mixed method APEASE evaluation of professional stakeholder results.

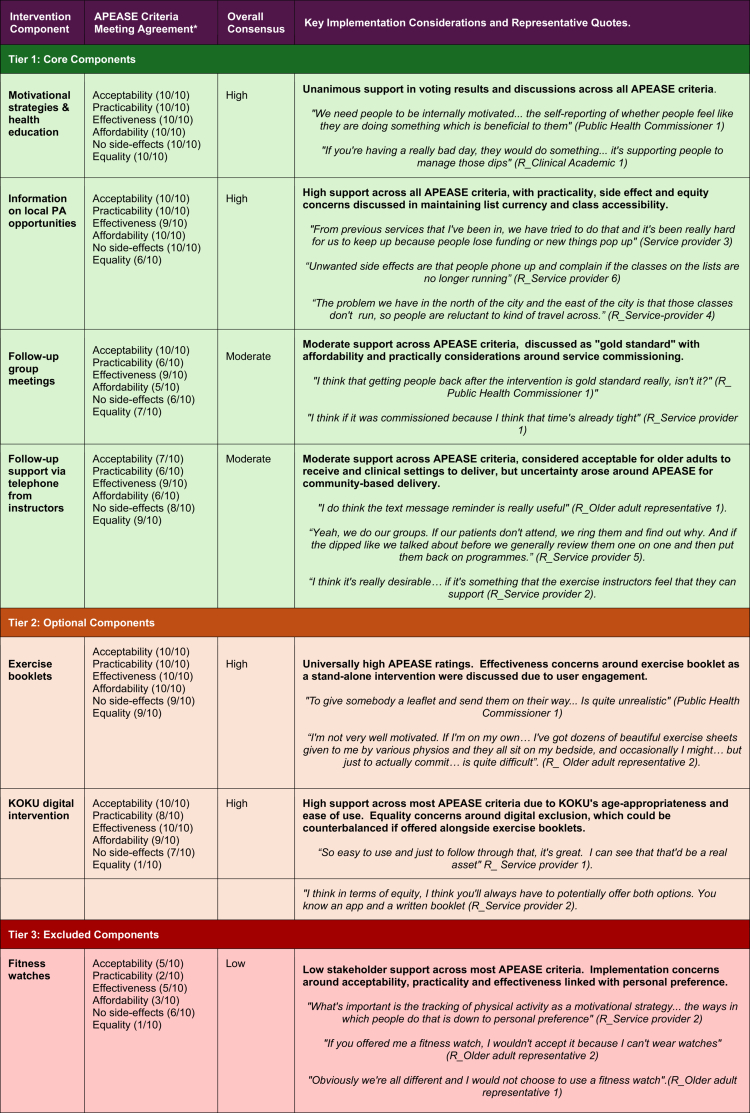

Three-Tiered Model based on APEASE criteria voting results. Numbers (n=) indicate the level of consensus in support of each intervention strategy for service-providers to deliver. Total professional stakeholder sample number 10. **Consensus classification**
High consensus: n ≥ 9 to 10 respondents across all APEASE criteria. Moderate consensus: *n* = 6 to 8 respondents across most criteria. Low consensus: n ≤ 5 respondents across most criteria.

### Stage 3: implementation planning results

**Stakeholders:** In stage 3, two service-providers (one PSI and one service manager) were invited and attended the implementation planning workshop.

**Coherence:** The hybrid model combining home-based exercise with monthly group follow-up aligned with existing FaME structures. However, KESS requires coordination with local community exercise providers for participant transitions, and availability of age-appropriate groups varies geographically across all four Healthworks sites. Service-providers noted that while two Healthworks sites could offer internal follow-on exercise services, two sites would need partnerships with external leisure centres for GP referral schemes.

**Collective action:** Sixty-minute sessions in familiar venues were deemed feasible with existing resources. Service-providers identified practical challenges around PSI-led clinical assessments, particularly time constraints within the 60-minute sessions, leading to adoption of the home-based FFMOT. The home-based Functional Fitness MOT (FFMOT) (De Jong et al., 2018; LaterLife Training, 2024) is a self-administered battery of functional tests compared to age- and sex-matched normative data. Home-based FFMOT results would inform feedback, goal setting and action-planning in subsequent group sessions. Group exercise provision suggested in Stage 2, was integrated into sessions in the form of short exercise snacks to avoid overloading session time. The schedule expanded from 6 to 8 sessions based on service-provider feedback about content volume and avoiding overloading participants.

**Cognitive participation:** Stakeholders viewed KESS as a valuable role extension that would support participant transitions from FaME services. Behaviour change theory and delivery, successful ageing education and FFMOT were identified as training needs, with particular emphasis on delivering ageing-related content in motivating ways. These needs were incorporated into the 2-day KESS training programme.

### Final KESS intervention

The multi-stage co-design process resulted in the rigorous development of the Keep Exercising and Stay Steady (KESS) programme: an 8-session group intervention delivered over 8 months by trained PSIs. The programme employs a hybrid delivery model with 3 sessions embedded within existing FaME 24-week programmes and 5 additional sessions delivered monthly over 6 months post- FaME completion (supplementary material 4 contains a TIDieR Checklist detailing the KESS intervention). Sessions are delivered in familiar community venues where original FaME programmes occurred, by the same PSIs.

**Programme Structure:** Each 60-minute session has a different focus but largely incorporates a variety of tailored behaviour change strategies, health education and exercise snacks delivered via a group motivational interviewing approach (Supplementary material 5).

### Core components

- Tailored motivational strategies including home-based FFMOT assessments, goal setting, relapse prevention, action-planning, self-monitoring activity, follow-up calls and text message reminders.
- Health behaviour education focusing on successful ageing, frailty avoidance, and reducing social isolation via community engagement activities.
- Tailored transition support to local PA opportunities and home-based exercise.
- Group-based social support and structured peer interaction activities.

### Optional components

- Choice between digital (KOKU digital intervention (Choi et al., 2021) via digital tablets) and traditional (illustrated booklets (LaterLife Training, 2024) home exercise support.
- Support for participants preferred PA monitoring tools.

### Implementation support

- Comprehensive 2-day training programme, collaboratively developed with a recognised not for profit training provider, a frailty specialist and a behaviour change researcher.
- Standard operating procedures and session-by-session implementation manuals.
- Participant resources including the LaterLife Training (LLT) Active Calendars (LaterLife Training, 2024), FFMOT at Home booklet (LaterLife Training, 2024), and local resources for PA opportunities. Participants were signposted to online social groups that promote PA habits, such as Make Movement Your Mission (Bosco et al., 2022).

## Discussion

This rigorous multi-stage co-design process successfully developed a theoretically grounded, stakeholder-informed intervention that bridges the gap between FaME programme completion and exercise maintenance. The co-design approach, increasingly recognised as essential in falls prevention research (McKercher et al., 2025; Morris et al., 2025), promotes intervention acceptability for both service-users and service-providers. The approach also addresses real-world implementation challenges that often impede intervention translation from research to practice (Leask et al., 2019).

High agreement across community and profession stakeholder groups demonstrates remarkable consensus regarding essential elements for exercise maintenance, challenging common assumptions about tensions between user wants and service constraints (Altman et al., 2018). Unanimous professional support for motivational strategies and education reflects a paradigm shift from traditional exercise prescription towards applied behaviour change approaches that address the complex interplay of individual, social, and environmental factors influencing PA (Maula et al., 2019; Pettersson et al., 2021; Yang et al., 2024). While education is effective in hospital falls prevention (Morris et al., 2022), evidence suggests that educational interventions alone may not sustain these benefits over time (Kempegowda et al., 2018), reinforcing our focus on strategies beyond initial education delivery.

Systematic review evidence shows that behavioural interventions can increase PA at 12 months in older adults, with tailored interventions incorporating transition support and goal setting appearing most promising (Hobbs et al., 2013; Stockwell et al., 2019). Exercise snacks and habit formation strategies also show promise for maintaining physical activity in later life (Du et al., 2025; Fleig et al., 2016; Karlsson et al., 2025). By practising exercise snacks during KESS sessions and encouraging participants to attach them to existing home routines (e.g. one leg balance whilst teeth brushing), the intervention transforms conscious exercise into automatic behaviour, addressing the common challenge of post-programme adherence (Finnegan et al., 2019; Pettersson et al., 2023). Integrating these evidence-based strategies, KESS addresses key factors for sustaining exercise beyond the initial PA intervention period where many interventions fail.

Information on local PA opportunities achieved high acceptability amongst professional stakeholders. Yet, community-based stakeholders emphasised transport accessibility, costs, and venue suitability issues that could exclude those with mobility limitations. These findings reflect broader evidence that older adults face compounded barriers to PA participation, including transport poverty and venue accessibility that information provision alone cannot address (Maula et al., 2019; Yang et al., 2024). This mirrors challenges identified in hospital-to-community transitions internationally (Hill et al., 2015; McLennan et al., 2024). The co-design approach adopted a pragmatic equity-focused solution, offering PA information alongside both digital and non-digital home-based exercise choices, maintaining choice to engage with different exercise options.

Our findings reveal a tension between digital intervention acceptability and equity concerns. While professional stakeholders rated digital interventions highly for effectiveness, they expressed strong concerns about widening inequalities, a paradox that reflects broader challenges in digital health implementation (Hepburn et al., 2025). Community stakeholders emphasised that digital inclusion requires ongoing support beyond technology provision, reflecting recognition that digital interventions may exclude older adults unless barriers are addressed (Wale et al., 2024). The KESS intervention addresses this through hybrid delivery (offering both digital and paper-based options) to accommodate different user preferences and abilities.

Community-based stakeholders emphasised the importance of maintaining social connections developed during FaME programmes. Although relatively few participants maintain exercise long-term, group-based falls prevention programmes can offer social support benefits to some attendees (Bunn et al., 2008; Lafond et al., 2019), with those maintaining PA levels post-programme reporting sustained social networks (Maula et al., 2019). The KESS intervention therefore combines opportunities for continued social engagement with behavioural strategies and flexible exercise options, recognising that exercise maintenance requires addressing both personal and community dimensions of physical activity.

## Methodological strengths and limitations

The multi-stage co-design approach incorporating diverse stakeholder perspectives, including the experiences of falls prevention programme completers whose perspectives may differ from dropouts and general older adult populations (Cornwall & Jewkes, 1995; McGowan et al., 2025) is the study’s primary strength. The co-design methodology, while ensuring local relevance and stakeholder engagement, inherently limits generalisability to other contexts which is a key limitation. While the intervention framework may be transferable, specific implementation procedures developed in Stage 3 may require substantial adaptation for different contexts (Murray et al., 2010). Despite reflecting typical falls prevention programme demographics, there was a high percentage of female and Caucasian service-users in Stage 1 which may limit representativeness of different perspectives, preferences and barriers to exercise maintenance. Addressing limitations may require additional consultation to ensure intervention appropriateness across different geographical areas, settings and stakeholder groups.

### Implications for practice, policy, research

KESS provides a replicable and scalable framework for exercise maintenance interventions. The co-design methods offer a template for adaptation to local contexts and needs. The tiered approach, balancing standardisation with personalisation presents a pragmatic model that is adaptable for other chronic disease management contexts. Professional stakeholder feedback underscores the systemic barriers to maintenance support within current funding structures. Policy makers should consider developing specific funding streams for maintenance interventions that bridge the gap between rehabilitation and long-term prevention.

Future research is needed to evaluate the KESS interventions acceptability, clinical and cost effectiveness and implementation potential across diverse service settings and population groups. Long-term follow-up would provide crucial evidence regarding intervention endurance and clinical significance. Economic evaluations incorporating implementation costs and healthcare utilisation are needed to inform policy decisions regarding maintenance intervention commissioning and sustainability.

## Conclusion

This multi-stage co-design process successfully developed an evidence-based exercise maintenance intervention addressing the critical gap when older adults exit FaME programmes. The intervention framework, informed by diverse stakeholder perspectives, is now ready for feasibility and acceptability evaluation.

## Supplementary Material

Audsley._Suppl_Materials._Final_Apr_26.docx Audsley._Suppl_Materials._Final_Apr_26.docx

## Data Availability

The datasets generated and analysed during the current study are not publicly available due to ethical restrictions to protect participant confidentiality, as participants did not consent to public data sharing. Aggregated data may be available from the corresponding author upon reasonable request.

## References

[cit0001] Altman, M., Huang, T. T., & Breland, J. Y. (2018). Design thinking in health care. *Preventing Chronic Disease*, *15*, E117. 10.5888/pcd15.180128 30264690 PMC6178900

[cit0002] Ambrose, A. F., Paul, G., & Hausdorff, J. M. (2013). Risk factors for falls among older adults: A review of the literature. *Maturitas*, *75*(1), 51–61. 10.1016/j.maturitas.2013.02.009 23523272

[cit0003] Audsley, S., Kendrick, D., Logan, P., Jones, M., & Orton, E. (2020). A randomised feasibility study assessing an intervention to keep adults physically active after falls management exercise programmes end. *Pilot and Feasibility Studies*, *6*, 37. 10.1186/s40814-020-00570-9 32161660 PMC7060620

[cit0004] Bosco, A., McGarrigle, L., Skelton, D. A., Laventure, R., Townley, B., & Todd, C. (2022). Make movement your mission: Evaluation of an online digital health initiative to increase physical activity in older people during the COVID-19 pandemic. *Digital Health*, *8*, 20552076221084468. 10.1177/20552076221084468 35295764 PMC8918976

[cit0005] Braun, V., & Clarke, V. (2006). Using thematic analysis in psychology. *Qualitative Research in Psychology*, *3*(2), 77–101. 10.1191/1478088706qp063oa

[cit0006] Bunn, F., Dickinson, A., Barnett-Page, E., McInnes, E., & Horton, K. (2008). A systematic review of older people's perceptions of facilitators and barriers to participation in falls-prevention interventions. *Ageing & Society*, *28*, 449–472. 10.1017/S0144686X07006861

[cit0007] Choi, N. G., Stanmore, E., Caamano, J., Vences, K., & Gell, N. M. (2021). A feasibility study of multi-component fall prevention for homebound older adults facilitated by lay coaches and using a tablet-based, gamified exercise application. *Journal of Applied Gerontology*, *40*(11), 1483–1491. 10.1177/0733464821991024 33541199 PMC8848472

[cit0008] Cornwall, A., & Jewkes, R. (1995). What is participatory research? *Social Science & Medicine*, *41*(12), 1667–1676. 10.1016/0277-9536(95)00127-S 8746866

[cit0009] Cunningham, C., O' Sullivan, R., Caserotti, P., & Tully, M. A. (2020). Consequences of physical inactivity in older adults: A systematic review of reviews and meta-analyses. *Scandinavian Journal of Medicine & Science in Sports*, *30*(5), 816–827. 10.1111/sms.13616 32020713

[cit0010] De Jong, L. D., Peters, A., Gawler, S., Chalmers, N., Henderson, C., Hooper, J., Laventure, R., McLean, L., & Skelton, D. (2018). The appeal of the functional fitness MOT to older adults and health professionals in an outpatient setting: A mixed-method feasibility study. *Clinical Interventions in Aging*, *13*, 1815–1829. 10.2147/CIA.S173481 30275688 PMC6156115

[cit0011] Du, Y., Peng, R., Wan, X., Zhang, C., Guo, Y., Chang, J., Feng, H., & Cao, Z. (2025). Perceptions and experiences of exercise snacks among middle‐aged and older adults: A systematic review and meta‐synthesis. *Public Health Nursing*, *42*(2), 1031–1046. 10.1111/phn.13495 39654268

[cit0012] Finnegan, S., Seers, K., & Bruce, J. (2019). Long-term follow-up of exercise interventions aimed at preventing falls in older people living in the community: A systematic review and meta-analysis. *Physiotherapy*, *105*(2), 187–199. 10.1016/j.physio.2018.09.002 30846193

[cit0013] Finnegan, S., Bruce, J., & Seers, K. (2019). What enables older people to continue with their falls prevention exercises? A qualitative systematic review. *BMJ Open*, *9*(4), e026074. 10.1136/bmjopen-2018-026074 PMC6500202 30992291

[cit0014] Fleig, L., McAllister, M. M., Chen, P., Iverson, J., Milne, K., McKay, H. A., Clemson, L., & Ashe, M. C. (2016). Health behaviour change theory meets falls prevention: Feasibility of a habit-based balance and strength exercise intervention for older adults. *Psychology of Sport and Exercise*, *22*, 114–122. 10.1016/j.psychsport.2015.07.002

[cit0015] Grindell, C., Coates, E., Croot, L., & O’Cathain, A. (2022). The use of co-production, co-design and co-creation to mobilise knowledge in the management of health conditions: A systematic review. *BMC Health Services Research*, *22*(1), 877. 10.1186/s12913-022-08079-y 35799251 PMC9264579

[cit0016] Harvey, J. A., Chastin, S. F., & Skelton, D. A. (2015). How sedentary are older people? A systematic review of the amount of sedentary behavior. *Journal of Aging and Physical Activity*, *23*(3), 471–487. 10.1123/japa.2014-0164 25387160

[cit0017] Hawley-Hague, H., Tacconi, C., Mellone, S., Martinez, E., Ford, C., Chiari, L., Helbostad, J., & Todd, C. (2020). Smartphone apps to support falls rehabilitation exercise: App development and usability and acceptability study. *JMIR Mhealth and Uhealth*, *8*(9), e15460. 10.2196/15460 32985992 PMC7551104

[cit0018] Heng, H., Jazayeri, D., Shaw, L., Kiegaldie, D., Hill, A., & Morris, M. E. (2020). Hospital falls prevention with patient education: A scoping review. *BMC Geriatrics*, *20*(1), 140. 10.1186/s12877-020-01515-w 32293298 PMC7161005

[cit0019] Hepburn, J., Williams, L., & McCann, L. (2025). Barriers to and facilitators of digital health technology adoption among older adults with chronic diseases: Updated systematic review. *JMIR Aging*, *8*, e80000. 10.2196/80000 40934502 PMC12464506

[cit0020] Hill, A.-M., McPhail, S. M., Waldron, N., Etherton-Beer, C., Ingram, K., Flicker, L., Bulsara, M., & Haines, T. P. (2015). Fall rates in hospital rehabilitation units after individualised patient and staff education programmes: A pragmatic, stepped-wedge, cluster-randomised controlled trial. *The Lancet*, *385*(9987), 2592–2599. 10.1016/S0140-6736(14)61945-0 25865864

[cit0021] Hobbs, N., Godfrey, A., Lara, J., Errington, L., Meyer, T. D., Rochester, L., White, M., Mathers, J. C., & Sniehotta, F. F. (2013). Are behavioral interventions effective in increasing physical activity at 12 to 36 months in adults aged 55 to 70 years? A systematic review and meta-analysis. *BMC Medicine*, *11*(1), 1–12. 10.1186/1741-7015-11-75 23506544 PMC3681560

[cit0022] Iliffe, S., Kendrick, D., Morris, R., Masud, T., Gage, H., Skelton, D., Dinan, S., Bowling, A., Griffin, M., Haworth, D., Swanwick, G., Carpenter, H., Kumar, A., Stevens, Z., Gawler, S., Barlow, C., Cook, J., & Belcher, C. (2014). Multicentre cluster randomised trial comparing a community group exercise programme and home-based exercise with usual care for people aged 65 years and over in primary care. *Health Technology Assessment (NIHR, Winchester, England)*, *18*(49), vii. 10.3310/hta18490 PMC4781144 25098959

[cit0023] James, E., Oman, P., Ali, M., Court, P., Goodall, S., Nichols, S. J., & O’Doherty, A. F. (2022). The effectiveness of the healthworks staying steady community-based falls prevention exercise programme to improve physical function in older adults: A 6-year service evaluation. *BMC Public Health*, *22*(1), 1457. 10.1186/s12889-022-13832-3 35915422 PMC9341056

[cit0024] Karlsson, Å., Lundell, S., Solbjør, M., & Pettersson, B. (2025). Staying active through life’s shifting seasons: A qualitative study of community-dwelling older adults’ experiences of habit formation and physical activity in later life. *European Review of Aging and Physical Activity*, *22*, 25. 10.1186/s11556-025-00393-8 41310451 PMC12670851

[cit0025] Kempegowda, P., Chandan, J. S., Hutton, R., Brown, L., Madden, W., Webb, J., Doyle, A., & Treml, J. (2018). Focused educational intervention improves but may not sustain knowledge regarding falls management. *BMJ Open Quality*, *7*(3), e000222. 10.1136/bmjoq-2017-000222 PMC6059340 30057952

[cit0026] Kwasnicka, D., Dombrowski, S. U., White, M., & Sniehotta, F. (2016). Theoretical explanations for maintenance of behaviour change: A systematic review of behaviour theories. *Health Psychology Review*, *10*(3), 277–296. 10.1080/17437199.2016.1151372 26854092 PMC4975085

[cit0027] Lafond, N., Maula, A., Iliffe, S., Vedhara, K., Audsley, S., Kendrick, D., & Orton, E. (2019). We got more than we expected.' older people's experiences of falls-prevention exercise interventions and implications for practice; a qualitative study. *Primary Health Care Research and Development*, *20*, e103. 10.1017/S1463423619000379 32800005 PMC6609972

[cit0028] LaterLife Training. (2024). LaterLife Training Resources. [cited 2024 20/10/2024]; Available from: https://laterlifetraining.co.uk/

[cit0029] Leask, C. F., Sandlund, M., Skelton, D. A., Altenburg, T. M., Cardon, G., Chinapaw, M. J. M., De Bourdeaudhuij, I., Verloigne, M., & Chastin, S. F. M. (2019). Framework, principles and recommendations for utilising participatory methodologies in the co-creation and evaluation of public health interventions. *Research Involvement and Engagement*, *5*(1), 2. 10.1186/s40900-018-0136-9 30652027 PMC6327557

[cit0030] Maula, A., LaFond, N., Orton, E., Iliffe, S., Audsley, S., Vedhara, K., & Kendrick, D. (2019). Use it or lose it: A qualitative study of the maintenance of physical activity in older adults. *BMC Geriatrics*, *19*(1), 349. 10.1186/s12877-019-1366-x 31830900 PMC6909612

[cit0031] May, C., & Finch, T. (2009). Implementing, embedding, and integrating practices: An outline of normalization process theory. *Sociology*, *43*(3), 535–554. 10.1177/0038038509103208

[cit0032] McAuley, E., Mullen, S. P., Szabo, A. N., White, S. M., Wójcicki, T. R., Mailey, E. L., Gothe, N. P., Olson, E. A., Voss, M., Erickson, K., Prakash, R., & Kramer, A. F. (2011). Self-regulatory processes and exercise adherence in older adults: Executive function and self-efficacy effects. *American Journal of Preventive Medicine*, *41*(3), 284–290. 10.1016/j.amepre.2011.04.014 21855742 PMC3160622

[cit0033] McGowan, L. J., Davies, A., French, D. P., Devereux‐Fitzgerald, A., Boulton, E., Todd, C., Phillipson, C., & Powell, R. (2025). Understanding the experiences of older adult participants and individuals involved in the delivery of a physical activity programme based on participatory approaches: A qualitative analysis. *British Journal of Health Psychology*, *30*(1), e12747. 10.1111/bjhp.12747 39313443 PMC11586820

[cit0034] McKercher, J. P., Peiris, C. L., Peterson, S., Thwaites, C., Hill, A., Clifford, A. M., & Morris, M. E. (2025). Consumer perspectives on implementing falls prevention and management in rehabilitation hospitals: Protocol for a qualitative study. *BMJ Open*, *15*(7), e101974. 10.1136/bmjopen-2025-101974 PMC12215128 40592754

[cit0035] McLennan, C., Sherrington, C., Tilden, W., Jennings, M., Richards, B., Hill, A., Fairbrother, G., Ling, F., Naganathan, V., & Haynes, A. (2024). Considerations across multiple stakeholder groups when implementing fall prevention programs in the acute hospital setting: A qualitative study. *Age and Ageing*, *53*(10), afae208. 10.1093/ageing/afae208 39354814 PMC11445322

[cit0036] Michie, S., Atkins, L., & West, R. (2014). *The behaviour change wheel: A guide to designing interventions*. London: Silverback Publishing.

[cit0037] Moll, S., Wyndham-West, M., Mulvale, G., Park, S., Buettgen, A., Phoenix, M., Fleisig, R., & Bruce, E. (2020). Are you really doing ‘codesign’? Critical reflections when working with vulnerable populations. *BMJ Open*, *10*(11), e038339. 10.1136/bmjopen-2020-038339 PMC7640510 33148733

[cit0038] Montero-Odasso, M., van der Velde, N., Martin, F. C., Petrovic, M., Tan, M. P., Ryg, J., Aguilar-Navarro, S., Alexander, N. B., Becker, C., Blain, H., Bourke, R., Cameron, I. D., Camicioli, R., Clemson, L., Close, J., Delbaere, K., Duan, L., Duque, G., Dyer, S. M., … Rixt Zijlstra, G. A. (2022). World guidelines for falls prevention and management for older adults: A global initiative. *Age and Ageing*, *51*(9), afac205. 10.1093/ageing/afac205 36178003 PMC9523684

[cit0039] Morris, M. E., Webster, K., Jones, C., Hill, A., Haines, T., McPhail, S., Kiegaldie, D., Slade, S., Jazayeri, D., Heng, H., Shorr, R., Carey, L., Barker, A., & Cameron, I. (2022). Interventions to reduce falls in hospitals: A systematic review and meta-analysis. *Age and Ageing*, *51*(5), afac077. 10.1093/ageing/afac077 35524748 PMC9078046

[cit0040] Morris, M. E., Said, C. M., Haines, T., Heng, H. W. F., Batchelor, F., Hutchinson, A. M., McKercher, J. P., Semciw, A. I., Hill, A., Peterson, S., Kane, R., Fowler-Davis, S., Campbell, S., Sherrington, C., Gilmartin-Thomas, J., Phan, U., & Thwaites, C. (2025). Reference standard for the prevention and management of hospital falls: A multidisciplinary delphi consensus study. *BMJ Open*, *15*(10), e105950. 10.1136/bmjopen-2025-105950 PMC12506041 41057179

[cit0041] Murray, E., Treweek, S., Pope, C., MacFarlane, A., Ballini, L., Dowrick, C., Finch, T., Kennedy, A., Mair, F., O'Donnell, C., Ong, B. N., Rapley, T., Rogers, A., & May, C. (2010). Normalisation process theory: A framework for developing, evaluating and implementing complex interventions. *BMC Medicine*, *8*(1), 1–11. 10.1186/1741-7015-8-63 20961442 PMC2978112

[cit0042] Orton, E., Audsley, S., Coupland, C., Gladman, J. R. F., Iliffe, S., Lafond, N., Logan, P., Masud, T., Skelton, D. A., Timblin, C., Timmons, S., Ward, D., & Kendrick, D. (2021). Real world' effectiveness of the falls management exercise (FaME) programme: An implementation study. *Age and Ageing*, *50*(4), 1290–1297. 10.1093/ageing/afaa288 33529311

[cit0043] Petersen, N., König, H.-H., & Hajek, A. (2020). The link between falls, social isolation and loneliness: A systematic review. *Archives of Gerontology and Geriatrics*, *88*, 104020. 10.1016/j.archger.2020.104020 32018091

[cit0044] Pettersson, B., Lundell, S., Lundin-Olsson, L., & Sandlund, M. (2023). Maintaining balance in life’—exploring older adults’ long-term engagement in self-managed digital fall prevention exercise. *European Review of Aging and Physical Activity*, *20*(1), 12. 10.1186/s11556-023-00322-7 37464299 PMC10354884

[cit0045] Pettersson, B., Janols, R., Wiklund, M., Lundin-Olsson, L., & Sandlund, M. (2021). Older adults’ experiences of behavior change support in a digital fall prevention exercise program: Qualitative study framed by the self-determination theory. *Journal of Medical Internet Research*, *23*(7), e26235. 10.2196/26235 34328438 PMC8367180

[cit0046] Rubenstein, L. Z. (2006). Falls in older people: Epidemiology, risk factors and strategies for prevention. *Age and Ageing*, *35*(suppl_2), ii37–ii41. 10.1093/ageing/afl084 16926202

[cit0047] Sherrington, C., Fairhall, N., Kwok, W., Wallbank, G., Tiedemann, A., Michaleff, Z. A., Ng, C. A. C. M., & Bauman, A. (2020). Evidence on physical activity and falls prevention for people aged 65+ years: Systematic review to inform the WHO guidelines on physical activity and sedentary behaviour. *International Journal of Behavioral Nutrition and Physical Activity*, *17*(1), 144. 10.1186/s12966-020-01041-3 33239019 PMC7689963

[cit0048] Sherrington, C., Fairhall, N. J., Wallbank, G. K., Tiedemann, A., Michaleff, Z. A., Howard, K., Clemson, L., Hopewell, S., & Lamb, S. E. (2019). Exercise for preventing falls in older people living in the communityCochrane Database of Systematic Reviews. 10.1002/14651858.CD012424.pub2. CD012424 PMC6360922 30703272

[cit0049] Skelton, D., Dinan, S., Campbell, M., & Rutherford, O. (2005). Tailored group exercise (Falls Management Exercise—FaME) reduces falls in community-dwelling older frequent fallers (an RCT). *Age and Ageing*, *34*(6), 636–639. 10.1093/ageing/afi174 16267192

[cit0050] Skelton, D. A., Rutherford, O. M., Dinan-Young, S., & Sandlund, M. (2019). Effects of a falls exercise intervention on strength, power, functional ability and bone in older frequent fallers: FaME (Falls Management Exercise) RCT secondary analysis. *Journal of Frailty Sarcopenia and Falls*, *4*(1), 11–19. 10.22540/JFSF-04-011 32300711 PMC7155371

[cit0051] Stockwell, S., Schofield, P., Fisher, A., Firth, J., Jackson, S. E., Stubbs, B., & Smith, L. (2019). Digital behavior change interventions to promote physical activity and/or reduce sedentary behavior in older adults: A systematic review and meta-analysis. *Experimental Gerontology*, *120*, 68–87. 10.1016/j.exger.2019.02.020 30836130

[cit0052] Tinetti, M. E., Speechley, M., & Ginter, S. F. (1988). Risk factors for falls among elderly persons living in the community. *New England Journal of Medicine*, *319*(26), 1701–1707. 10.1056/NEJM198812293192604 3205267

[cit0053] Ventre, J. P., Manning, F., Mahmoud, A., Brough, G., Timmons, S., Hawley-Hague, H., Skelton, D. A., Goodwin, V. A., Todd, C. J., Kendrick, D., Logan, P., & Orton, E. (2025). Factors influencing fall prevention programmes across three regions of the UK: The challenge of implementing and spreading the falls management exercise (FaME) programme in a complex landscape. *Age and Ageing*, *54*(4), afaf083. 10.1093/ageing/afaf083 40207379 PMC11982667

[cit0054] Wale, A., Everitt, J., Ayres, T., Okolie, C., Morgan, H., Shaw, H., Tudor Edwards, R., Davies, J., Lewis, R., Cooper, A., & Edwards, A. (2024). *A rapid review of the effectiveness of interventions for addressing digital exclusion in older adults* Health and Care Research Wales Evidence Centre. 10.2196/70377 PMC12826648 41468583

[cit0055] Xu, Z., Shamsulariffin, S., Azhar, Y., & Xi, M. (2025). Does self‐determination theory associate with physical activity? A systematic review of systematic review. *International Journal of Psychology*, *60*(3), e70044. 10.1002/ijop.70044 40256835

[cit0056] Yang, Y., Gao, Y., An, R., & Wan, Q. (2024). Barriers and facilitators to exercise adherence in community-dwelling older adults: A mixed-methods systematic review using the COM-B model and theoretical domains framework. *International Journal of Nursing Studies*, *157*, 104808. 10.1016/j.ijnurstu.2024.104808 38823146

[cit0057] Yardley, L., Ainsworth, B., Arden-Close, E., & Muller, I. (2015). The person-based approach to enhancing the acceptability and feasibility of interventions. *Pilot and Feasibility Studies*, *1*, 37. 10.1186/s40814-015-0033-z 27965815 PMC5153673

