# Supplementary material for: Co-designing an exercise maintenance intervention for older adults exiting falls prevention programmes: a multi-stage stakeholder consultation approach
Source: Health Psychol Behav Med. 2026 Apr 22;14(1):2662110. doi: 10.1080/21642850.2026.2662110 (PMC13103995; doi:10.1080/21642850.2026.2662110)
Supplement: Audsley._Suppl_Materials._Final_Apr_26.docx [file RHPB_A_2662110_SM5103.docx]

**Supplementary Material 1.**

**Table 1: Thematic analysis and representative quotes outlining community-based stakeholder group discussions.**

| **Theme** | **Sub-themes** | **Representative Quotes** |
| --- | --- | --- |
| **Providing Information on Local PA Opportunities** | - Information essential but maintaining currency resource-intensive - Practical details needed (transport, cost, accessibility) - Personalized matching challenging | *"If you haven't got all the information, you're not going to know where to go, are you?" (R6_Service user)*  *"Is it on a bus route. Can people get there. But also, the cost as well"*  *(R4_Service user)*  *“Keeping on top of that list would be a significant part of our job"*  *(R9_Professional)*  *"it's picking the right thing for the right person... that's quite challenging sometimes." (R1_Professional)*  *““I do think we each have to discover for ourselves whether it’s a safe space for us“ (R4_service user)* |
| **Delivering Motivational Strategies** | - Peer support and scheduled accountability valued - Flexibility in goal-setting to avoid failure - Contingency planning for high-risk situations | *"Having somebody... if we said we were going to meet once a week, or once a month, it would be good" (R2_Service user)*  *“Not being too rigid in your goals... if you don't achieve it once, you're like, oh well, failed" (R6_Service user)*  *"Having, like, contingencies... what are we going to do if this class doesn't run anymore?" (R6_Professional)* |
| **Follow-up Group Meetings** | - Social connection/loneliness reduction as primary value - Peer accountability motivates exercise engagement - Valued for social contact regardless of exercise content | *"I've talked to most of the people in this class... they are actually lonely" (R6_Service user)*  *“I think it would be great, as you mentioned... Whether it would encourage me to exercise, I don’t know. But it would be nice to meet people." (R4_Service user)*  *" It would encourage you to exercise, because you would be wanting to, like, report back what you’d done” (R9_Service user)* |
| **Providing Health Education** | - Universally valued but effectiveness questioned - Preference for accessible instructor delivery over medical complexity | *"I think all education is good." (R1_Service user)*  *“My doctor tends to send me a ton of stuff that bamboozles me... I tend to ask my class instructor" (R2_Service-user)*  *“I think the education is important. I'm not sure this is going to be the most effective method" (R4_Service user)*  *“I know I talk about, kind of, the guidelines during my education session. …But I guess it’s probably needs going into more depth, doesn’t it?”* *(R6_Professional)* |
| **Technology-based vs paper-based interventions for home exercise** | **Home Exercise via KOKU digital intervention**   - Simplicity and progress tracking as key benefits - Digital literacy variable with ongoing support needed - Visual demonstration superior to written instructions | *"It's simple (KOKU)... that’s what I really like about it … you can see how much you've progressed" (R1_Service user)*  *“I said I was no good with technology, but watching that (KOKU) is a lot better than written” (R6 _Service user)*  *"I think people are more likely to have phones" (R4_Service user)*  *"Not everybody has got access to technology... Some people will thrive, and others are just left" (R7_Professional)*  *“If you had support … show you how to use it and if that (support) was ongoing” (R5_Service-user)* |
|  | **Home Exercise via Exercise Booklet**   - High usage but static images limiting - Language accessibility concerns - Complementary to digital options | *"I used it... I use it a lot" (R5_Service user)*  *"You need to see the exercise to copy it, don't you?" (R6_service-user)*  *"My only concern is we had a lovely elderly Asian lady... she couldn't read the English" (R1_Service user)*  *“I mean, the good thing about the App is that you can see the movement. Whereas obviously a booklet is just a still photograph. So, maybe having both” (R9_Professional)* |
|  | **Fitness Watch Provision**   - Motivational value highly individual - Alternative functions valued (fall detection) | *"If I get this watch, that would add to my steps... I would love that, the motivation bit" (R4_Service user)*  *“It doesn't motivate me at all... Just being honest" (R1_Service user)*  *"The main reason I've got this was because of falling... I can phone them" (R1_Service user)*  ***“****Well, I think your watch is part of a motivational strategy****”****. (R3_Professional)* |
| **Staying Connected via Mobile Phone** | - Authentic relationships preferred over forced connections - Information governance concerns for professionals | *"I think you only want to swap phone numbers with people you've got friendly with" (R1_Service user)*  *"WhatsApp is quite easy – once you've got it installed" (R2_Service user)*  *“ What's the kind of rules around sharing phone numbers... I can imagine that's a bit of a minefield" (R9_Professional)* |
| **Cross-Cutting Themes:**   - Personalisation essential for acceptability - Digital inclusion requires active support - Social connection valued equally to exercise outcomes - Practical accessibility beyond information provision - Professional resource capacity concerns. | | |

**Legend.** PA, Physical Activity; KOKU, Keep On Keep Up.  **Note:** Table 1: Participants identified by respondent number and role.

**Supplementary Material 2.**

**Table 2: Stakeholder group voting results**

| **Intervention component** | **APEASE Criteria** | **Agree n (%)** | **Disagree n (%)** | **Total n** |
| --- | --- | --- | --- | --- |
| **Follow-up group meetings** | Acceptable to providers | 10 (100) | 0 (0) | 10 |
|  | Practical to deliver | 6 (60) | 4 (40) | 10 |
|  | Effective for users | 9 (90) | 1 (10) | 10 |
|  | Affordable for providers | 5 (50) | 5 (50) | 10 |
|  | Affordable for users | 9 (90) | 1 (10) | 10 |
|  | No side effects on providers | 6 (60) | 4 (40) | 10 |
|  | No side effects on users | 9 (90) | 1 (10) | 10 |
|  | Promotes equality | 7 (70) | 3 (30) | 10 |
| **Motivational strategies & health education** | Acceptable to providers | 10 (100) | 0 (0) | 10 |
|  | Practical to deliver | 10 (100) | 0 (0) | 10 |
|  | Effective for users | 10 (100) | 0 (0) | 10 |
|  | Affordable for providers | 10 (100) | 0 (0) | 10 |
|  | Affordable for users | 10 (100) | 0 (0) | 10 |
|  | No side effects on providers | 10 (100) | 0 (0) | 10 |
|  | No side effects on users | 10 (100) | 0 (0) | 10 |
|  | Promotes equality | 10 (100) | 0 (0) | 10 |
| **Information on local PA opportunities** | Acceptable to providers | 10 (100) | 0 (0) | 10 |
|  | Practical to deliver | 10 (100) | 0 (0) | 10 |
|  | Effective for users | 9 (90) | 1 (10) | 10 |
|  | Affordable for providers | 10 (100) | 0 (0) | 10 |
|  | Affordable for users | 10 (100) | 0 (0) | 10 |
|  | No side effects on providers | 9 (90) | 1 (10) | 10 |
|  | No side effects on users | 10 (100) | 0 (0) | 10 |
|  | Promotes equality | 6 (60) | 4 (40) | 10 |
| **KOKU Digital Intervention** | Acceptable to providers | 10 (100) | 0 (0) | 10 |
|  | Practical to deliver | 8 (80) | 2 (20) | 10 |
|  | Effective for users | 10 (100) | 0 (0) | 10 |
|  | Affordable for providers | 9 (90) | 1 (10) | 10 |
|  | Affordable for users | 9 (90) | 1 (10) | 10 |
|  | No side effects on providers | 9 (90) | 1 (10) | 10 |
|  | No side effects on users | 7 (70) | 3 (30) | 10 |
|  | Promotes equality | 1 (10) | 9 (90) | 10 |
| **Exercise booklets** | Acceptable to providers | 10 (100) | 0 (0) | 10 |
|  | Practical to deliver | 10 (100) | 0 (0) | 10 |
|  | Effective for users | 10 (100) | 0 (0) | 10 |
|  | Affordable for providers | 10 (100) | 0 (0) | 10 |
|  | Affordable for users | 10 (100) | 0 (0) | 10 |
|  | No side effects on providers | 9 (90) | 1 (10) | 10 |
|  | No side effects on users | 8 (80) | 2 (20) | 10 |
|  | Promotes equality | 9 (90) | 1 (10) | 10 |
| **Fitness watches** | Acceptable to providers | 5 (50) | 5 (50) | 10 |
|  | Practical to deliver | 2 (20) | 8 (80) | 10 |
|  | Effective for users | 5 (50) | 5 (50) | 10 |
|  | Affordable for providers | 3 (30) | 7 (70) | 10 |
|  | Affordable for users | 3 (30) | 7 (70) | 10 |
|  | No side effects on providers | 6 (60) | 4 (40) | 10 |
|  | No side effects on users | 6 (60) | 4 (40) | 10 |
|  | Promotes equality | 1 (10) | 9 (90) | 10 |
| **Follow-up phone calls** | Acceptable to providers | 8 (80) | 2 (20) | 10 |
|  | Practical to deliver | 7 (70) | 3 (30) | 10 |
|  | Effective for users | 9 (90) | 1 (10) | 10 |
|  | Affordable for providers | 6 (60) | 4 (40) | 10 |
|  | Affordable for users | 10 (100) | 0 (0) | 10 |
|  | No side effects on providers | 7 (70) | 3 (30) | 10 |
|  | No side effects on users | 8 (80) | 2 (20) | 10 |
|  | Promotes equality | 9 (90) | 1 (10) | 10 |

**PA, Physical Activity; KOKU, Keep On Keep Up; n, number of responses.**

**Supplementary Material 3.**

**Table 3: Thematic analysis professional stakeholder group discussions**

| **Theme** | **Sub-Themes** | **Representative Quotes** |
| --- | --- | --- |
| **Information on Local PA Opportunities** revealed universal recognition of the importance of providing people with information. Yet, significant implementation challenges were highlighted around maintaining information currency and providing tailored advice. | - Universal importance but maintaining currency challenging - Information Now website preferred but reliability issues - Tailored advice crucial but resource-intensive - Transition barriers (transport, cost, accessibility) - Waiting lists create "stress points" | *"From previous services that I've been in, we have tried to do that and it's been really hard for us to keep up because people lose funding or new things pop up" (R_Service provider 3)*  *“the unwanted side effects are that people phone up and complain if the classes on the lists are no longer running” (R_Service provider 6)*  *“the problem we have in the north of the city and the east of the city is that those classes don't run, so people are reluctant to kind of travel across.”* *(R_Service provider 4)*  *"It's completely unrealistic for us to kind of double check everything for them" (R_Service provider 7)* |
| **Follow-up Group Meetings** were viewed as the "gold standard" but raised concerns about seeking commissioning and funding support. | - "Gold standard" but requires commissioning and funding - Timing important (introduce month 4) - Content should balance education, behavior change, and exercise; | *"I think that getting people back after the intervention is gold standard really, isn't it?" (R_Public Health Commissioner 1)*  *"Once they're like four months in, they're like, OK, what are we going to do when this finishes?" (R_Service provider 7)*  *"I think if it was commissioned because I think that time's already tight" (R_Service provider 1)* |
| **Motivational Strategies and Health Education** were considered essential, with emphasis on tailored behaviour change approaches, personalised goal setting, and relapse prevention. Clinical assessments with feedback were considered important but noted as time-consuming. | - Essential with tailored behavior change and personalised goals critical - Relapse prevention and managing "dips" important - Functional fitness assessments motivating but time-consuming - Motivational interviewing recommended for standardisation. | *"We need people to be internally motivated... the self-reporting of whether people feel like they are doing something which is beneficial to them" (R_Public Health Commissioner 1)*  *"If you're having a really bad day, they would do something... it's supporting people to manage those dips" (R_Clinical Academic 1)*  *“It takes a lot of time to do the functional outcome assessment, so you'd have to be very savvy about what you chose and chose something simple.” (R_Clinical Academic 1)* |
| **Fitness Watches & PA Monitoring** faced questions about acceptability and affordability, with stakeholders favouring support for individuals' preferred tracking methods rather than universal device provision. | - Acceptability and affordability questioned - Wearability concerns and physical limitations - Emphasis on supporting preferred tracking methods rather than device provision - Tracking strategy more important than device type | *"I would think that what's important is the tracking of physical activity as a motivational strategy... the ways in which people do that is down to personal preference" (R_Service provider 2)*  *"If you offered me a fitness watch, I wouldn't accept it because I can't wear watches" (R_Older adult representative 2)*  *"It doesn't necessarily have to be one size fits all" (Service provider 5)* |
| **Exercise Booklet** was deemed insufficient alone, requiring personalisation and tailored prescriptions linked to individual achievement goals for effectiveness. | - Insufficient alone without personalisation - Must link to individual goals and tailored prescriptions - Need for progression to maintain challenge - Should be offered alongside digital options for equity | *"to give somebody a leaflet and send them on their way... Is quite unrealistic" (R_Public Health Commissioner 1)*  *"What we shouldn't be doing is going… here's the exercise booklet" (R_Clinical Academic 1)*  *"Those exercises have been completely linked to what they want to achieve" (R_Clinical Academic 1)* |
| **KOKU Digital Intervention** received positive reception due to age-appropriateness and ease of use, with digital support and peer connectivity features viewed as enhancing effectiveness. | - Age-appropriateness and ease of use valued - Digital support and peer connectivity enhance effectiveness - Introducing within programme helps overcome resistance - Ongoing technical support needed | *"So easy to use and just to follow through that, it's great... I would definitely use it" (R_Service provider 1)*  *"This is an age appropriate app really" (R_Service provider 2)*  *"If I'm connected to XX (via KOKU) and saying, oh, look, I did this today... That's much more likely to keep me going" (R_Older adult representative 1)*  *I think it looks fab by the way, the (KOKU) app. I think in terms of equity, I think you'll always have to potentially offer both options. You know an app and a written booklet (R_Service provider 2).* |
| **Follow-Up Support** through text reminders and telephone contacts was viewed as highly acceptable and practical, despite acknowledged resource intensity. | **Text / phone instructor follow-up**   - Highly acceptable despite resource intensity - Phone calls "desirable" and enable motivational coaching - Important given life changes happen quickly - Text reminders valued for diary management | *"People love a phone call, don't they?... doing a bit of the motivational coaching approach would probably be quite a good idea" (R_Service provider 1)*  *“I think it's really desirable if, as you say, if it's, if it's something that the exercise instructors feel that they can support …..A lot can happen in an older person's life in a month. So I think if somebody doesn't come to a group, it is really nice... to follow up" (R_Service provider 2)*  *"I do think the text message reminder is really useful" (R_Older adult representative 1)* |
| **Group Exercise Provision** was suggested as a new strategy as it was believed to improve intervention acceptability and enhance motivation for exercise. | - Exercise component improves acceptability - "Missed opportunity" not to include activity | *“that would be a missed opportunity if you didn't continue to do some activity at that session as well.” (R_Public Health Commissioner 1)*  *“I'm just wondering if there isn't an exercise component within that, how well that would be received?” (R_Service_ provider 2)* |
| **Cross-Cutting Themes:**   - Personalisation essential - Commissioning and resource sustainability concerns - Individual choice over one-size-fits-all - Balance between standardisation and local adaptation needed | | |

**Legend:** PA, Physical Activity; KOKU, Keep On Keep Up. **Note:** Table 3: Participants identified by stakeholder role and number.

**Supplementary Material 4.**

**Table 4: Complete TIDieR Checklist for the Keep Exercising & Stay Steady (KESS) Intervention**

Table 4 provides a comprehensive description of the KESS intervention using the TIDieR (Template for Intervention Description and Replication) checklist.

| 1. BRIEF NAME | Keep Exercising & Stay Steady (KESS) Intervention - A behaviour change intervention embedded within and following FaME programmes to support physical activity maintenance in older adults. | |
| --- | --- | --- |
| 2. WHY  (rationale, theory, or goal of the intervention) | **Rationale:** Physical activity levels decrease 6-24 months after FaME programmes end as older adults revert to old behaviours. Continuing strength and balance exercise is critical to maintain health gains, functional status, and reduced falls risk.  **Theoretical basis:** Social Cognitive Theory & Self-Determination Theory  **Goal:** Support older adults to develop positive and long-lasting physical activity behaviours after falls prevention exercise programmes end. | |
| 3. WHAT -  Materials | **Participant Materials:**   - Functional Fitness MOT (FFMOT) at-home handbook - Home exercise booklets (illustrated) - LLT Active Calendar - KOKU digital falls prevention exercise intervention on tablets - Information Now leaflets and website access - Make Exercise Your Mission Facebook page - Local physical activity timetable - Community-based physical activity information | **Provider Materials:**   - PSI KESS training manual - Standard Operating Procedures document - Session-specific scripts (1-24) - Digital equipment for older adult loan |
| 4. WHAT - Procedures | **Eight sessions over 9 months:**  **Sessions 1-3 (embedded in final 12 weeks of FaME):**   - Session 1: Self-assessing functional fitness using FFMOT - Session 2: Education on physical activity options and local opportunities; home exercise options - Session 3: Goal setting and action planning for physical activity and creating social support networks   **Sessions 4-8 (post-FaME, over 6 months):**   - Session 4 (1 month): Behaviour monitoring; identifying barriers/facilitators; developing "rainy day" plans - Session 5 (2 months): Reviewing FFMOT scores, goals, and action plans - Session 6 (3 months): Education on successful ageing; creating holistic action plans - Session 7 (4 months): Building social support networks; community connections - Session 8 (6 months): Final progress review; future planning; celebration | **Behaviour Change Strategies:**   - Self-assessment and monitoring - SMART goal setting (outcome and behaviour focused) - Action planning ("what, where, when, who with") - Relapse prevention ("rainy day" plans) - Self-monitoring (calendars, logs, apps, fitness watches) - Social support development - Motivational interviewing (OARS principles) - Exercise snacks with progressions   **Support Activities:**   - Text message reminders for sessions - Follow-up phone calls if sessions missed - Equipment loans (tablets, fitness watches) |
| 5. WHO PROVIDED (expertise, background and any specific training) | **Expertise/Background:**   - Postural Stability Instructor (PSI) - Knowledge of safe and effective exercise prescription for falls prevention populations | **Specific Training Provided (6 hours):**   - Functional Fitness MOT (FFMOT) administration - Behaviour change theory and application - Motivational interviewing communication methods - Physical activity and successful ageing education - Digital exercise options (KOKU digital intervention, fitness watches, videos) - Intervention protocol and data collection procedures |
| 6. HOW  (modes of delivery) | **Primary Mode:** Face-to-face group sessions  **Supplementary Modes:**   - Telephone follow-up (if unable to attend face-to-face) - One-to-one telephone support available if face-to-face attendance not possible - Text message reminders - Digital/app-based (KOKU digital intervention, Facebook videos) - Written materials (booklets, calendars) | |
| 7. WHERE (types of locations, infrastructure/ features) | **Venue:** Community-based settings at HealthWorks (Newcastle Upon Tyne, UK)  **Infrastructure Requirements:**   - Space suitable for group discussions and exercise practice - Safe environment for strength and balance exercises - Chairs and stable surfaces for exercise demonstrations - Projector/screen for educational presentations - Fire exits, first aid facilities, appropriate lighting | **Home Environment:**   - Participants perform self-assessments and home exercises - Safety requirements: stable floor surface, free from hazards, good lighting, stable chair, kitchen bench for support |
| 8. WHEN and HOW MUCH | **Total Duration:** 9 months  **Number of Sessions:** 8 group sessions  **Session Duration:** Approximately 60 minutes (including exercise and education components)  **Home Exercise Dose:** Participants encouraged to perform strength and balance exercise 3 times per week (30 minutes per session) using KOKU digital intervention, exercise booklets, or other options. CMO Physical Activity Recommendations encouraged. | |
| 9. TAILORING | **Personalisation Elements**  **What:** Goals, action plans, exercise options, monitoring tools, social support strategies  **Why:** To ensure intervention is meaningful, achievable, and relevant to individual circumstances, preferences, and functional abilities  **When:** Throughout intervention, particularly Sessions 3, 4, 5, and 8  **How:**   - **FFMOT Self-Assessment:** Participants assess own functional fitness; scores guide personalised exercise recommendations - **SMART Goal Setting:** Person-centred goals based on individual FFMOT scores, functional needs, and meaningful outcomes - **Action Planning:** "What, where, when, who with" approach tailored to individual circumstances - **Exercise Options:** Multiple formats offered (KOKU digital intervention, booklets, local classes, Facebook videos), participants choose preferred methods - **Monitoring Tool Selection:** Participants select preferred tracking method (calendar, diary, fitness watch, phone app) - **Rainy Day Plans:** Individual barrier identification and personalised coping strategies - **Exercise Progression:** KOKU digital intervention automatically progresses based on individual ability; FFMOT re-assessment guides modifications - **Motivational Interviewing:** PSIs use open questions, reflective listening to elicit individual motivations, barriers, and solutions | |
| 10. MODIFICATIONS | *Modifications during delivery would be documented in the final study report once the intervention has been delivered and evaluated.* | |
| 11. HOW WELL - Planned  (adherence or fidelity strategies and assessments by whom) | **Adherence Assessment:**  **Session Attendance:**  • Attendance rates to in-person and telephone sessions tracked on class registers maintained by PSIs  **Exercise Engagement:**  • Physical Activity Scale for the Elderly (PASE) questionnaire completed at baseline and post-intervention | **Fidelity Strategies:**   - **Standard Operating Procedures:** Detailed session-by-session guidance - **Scripted Content:** 24 scripts provided for key communication points - **PSI Training:** structured training package with learning outcomes - **Session Checklists:** Structured session plans with step-by-step procedures - **Materials Standardisation:** All participants receive same core materials - **Text Reminders:** Automated session reminders to support attendance - **Follow-up Calls:** Systematic contact for missed sessions |
| 12. HOW WELL - Actual | *Intervention adherence will be documented in the final study report once the intervention has been delivered and evaluated.* | |

**Legend: PA,** Physical Activity; **KOKU,** Keep On Keep Up; **LLT,** Later Life Training**; FaME,** Falls Management Exercise; **SMART,** Specific, Measurable, Achievable, Relevant, Time-bound; **PSI,** Postural Stability Instructor; **FFMOT,** Functional Fitness MOT**; CMO,** Chief Medical Officer; **OARS,** Open questions, Affirmations, Reflective listening, Summarising; **MI,** Motivational Interviewing; **PASE,** Physical Activity Scale for the Elderly**.**

**Supplementary Material 5.**

**Table 5: Detailed KESS intervention content and schedule of activities.**

| **Session 1: Self-Assessing Functional Fitness**  Embedded in Falls Management Exercise (FaME) Programme*: 10-12 weeks before FaME ends* | |
| --- | --- |
|  |  |
| **Session Content** | Welcome and health/safety procedures |
|  | Give education on importance of functional fitness |
|  | Provide Functional Fitness MOT (FFMOT) at Home handbook and explain purpose/process |
|  | Demonstrate each FFMOT assessment |
|  | Ask participants to practice assessments safely |
|  | Instruct on home FFMOT performance with booklet & safety advice |
|  | Introduce score comparison charts |
|  | Advise logging scores |
|  | Deliver strength and balance exercise (40 min) |
| **Session 2: Planning to Keep Exercising**  Embedded in FaME Programme*: 8 weeks before FaME ends* | |
|  |  |
| **Session Content** | Welcome and explain session focus on PA maintenance |
|  | Educate on PA guidelines and sedentary behaviour reduction |
|  | Use motivational interviewing (MI) to explore current PA behaviours |
|  | Group review of FFMOT at Home outcomes and meaning of scores in relation to function/falls |
|  | Provide information on local PA classes |
|  | Demonstrate "Information Now" website navigation |
|  | Enquire about current home exercise engagement |
|  | Introduce exercise booklet with specific exercises |
|  | Demonstrate KOKU digital intervention functionality and features |
|  | Show Make Movement Your Mission Facebook and You Tube pages <https://www.youtube.com/channel/UCqen30veJkDw_izbDFMyb6w> |
|  | Ask participants to consider goals and exercise options |
| **Session 3: Turning Plans into Actions**  Embedded in FaME Programme*: Final FaME session* | |
|  |  |
| Session Content | Welcome and deliver exercise session (30 min) |
|  | Introduce LLT Active Calendar with goal icons |
|  | Facilitate reflection on FFMOT scores and PA preferences |
|  | Establish person-centred meaningful long-term goals |
|  | Set SMART short-term behavioural goals |
|  | Develop weekly action plans ("what, where, when, who") |
|  | Include enjoyable activities in plans |
|  | Create "rainy-day" substitute action plans |
|  | Brainstorm social support mechanisms |
|  | Encourage exercise buddy partnerships |
|  | Provide KESS session information and text reminders |
|  | Close with summary and calendar planning |
| **Session 4: Monitoring & Rainy-Day Plans**  Post-FaME Follow-up*: 1 month after FaME ends* | |
|  |  |
| Session Content | Welcome and introduce session on staying on track |
|  | Educate on behaviour change as non-linear process |
|  | Identify personal PA barriers and high-risk situations |
|  | Problem-solve realistic barrier solutions in small groups |
|  | Create "what if" plans for overcoming barriers |
|  | Develop "getting back on track" plans |
|  | Educate on a range of PA monitoring tools (benefits/drawbacks) |
|  | Demonstrate various tracking methods (diaries, apps, watches) |
|  | Choose preferred monitoring method with action plan |
|  | Deliver exercise snacks |
|  | Close with reinforcement and reminder to do re-do home FFMOT test |
| **Session 5: Reviewing Outcomes & Plans**  **Post-FaME Follow-up:** *2 months after FaME ends* | |
|  |  |
| Session Content | Welcome and explain focus on progress review |
|  | Review FFMOT re-assessment scores and improvements |
|  | Compare past and present scores with written notes |
|  | Provide reassurance about maintaining vs improving |
|  | Use social comparison to increase motivation |
|  | Reflect on PA behaviours connecting to FFMOT outcomes |
|  | Review monitoring tool outcomes and progress |
|  | Assess if doing enough strength/balance exercise |
|  | Review goal attainment and action plan suitability |
|  | Modify goals/plans if needed for relevance |
|  | Deliver exercise snacks |
|  | Close with calendar logging encouragement |
| **Session 6: Skilling Up to Age Well**  **Post-FaME Follow-up***: 3 months after FaME ends* | |
|  |  |
| Session Content | Welcome and introduce ageing well focus |
|  | Show Okinawa society YouTube video for social comparison <https://youtu.be/39ce8WA6mSM> |
|  | Facilitate dialogue on successful ageing factors |
|  | Confirm successful ageing components |
|  | Educate on frailty avoidance behaviours (including PA, nutrition, cognitive stimulation, social interaction) |
|  | Create personalised successful ageing action plans, encouraging behaviours beyond PA |
|  | Deliver exercise snacks |
|  | Close with social support and community information preview |
| **Session 7: Building Active Communities**  **Post-FaME Follow-up***: 4 months after FaME ends* | |
|  |  |
| Session Content | Welcome and introduce social support/community focus |
|  | Outline social support types (emotional, practical, etc.) |
|  | Explore current methods of staying connected |
|  | Discuss group connectivity between KESS sessions |
|  | Provide information on local and age-appropriate community groups |
|  | Identify people who provide social support |
|  | Plan how people can support PA plans |
|  | Creating plans to meet and be active with others |
|  | Deliver exercise snacks |
|  | Close with final session preparation instructions |
| **Session 8: Future Self-Management**  **Post-FaME Follow-up***: 6 months after FaME ends* | |
|  |  |
| Session Content | Welcome to final session focusing on progress/future |
|  | Deliver exercise snacks |
|  | Provide completion certificates with active pledge |
|  | Encourage reflection on behaviours and 6-month outcomes |
|  | Review FFMOT scores across programme |
|  | Reflect on goal achievements and PA engagement |
|  | Reflect on confidence in daily/recreational activities |
|  | Share progress stories in group setting |
|  | Review most useful behavioural strategies |
|  | Plan future PA maintenance using preferred behavioural strategies |
|  | Set new goals for post-KESS period |
|  | Arrange peer check-ins for ongoing goal support |
|  | Reflect on social activity benefits |
|  | Encourage ongoing community group participation |
|  | Close with refreshments |

**Legend: PA:** Physical Activity; **FaME:** Falls Management Exercise;  **FFMOT:** Functional Fitness MOT; **MI:** Motivational Interviewing; **LLT:** Later Life Training
